# Metacontrol is reflected in phasic but not tonic cognitive control dynamics

**DOI:** 10.1038/s41598-025-20479-8

**Published:** 2025-09-24

**Authors:** Xi Wang, Xianzhen Zhou, Christian Beste, Bernhard Hommel

**Affiliations:** 1 Cognitive Neurophysiology, Department of Child and Adolescent Psychiatry, Faculty of Medicine, TU Dresden, Fetscherstrasse 74, 01307 Dresden, Germany; 2 School of Psychology, Shandong Normal University, Jinan, China

**Keywords:** Cognitive control, Metacontrol, EEG, Aperiodic activity, Creativity, Human behaviour, Medical research

## Abstract

Metacontrol, the regulation of cognitive strategies to balance persistence and flexibility, has been theorized to operate either as a tonic state, maintaining a consistent neural bias, or as a phasic mechanism, dynamically adjusting to situational demands. This study tested these scenarios by examining the relationship between metacontrol biases and changes in the aperiodic exponent of EEG activity. Behavioral results replicated well-established effects. Neural analyses revealed significant post-stimulus increases in the aperiodic exponent during incongruent trials, reflecting enhanced persistence in response to high-conflict conditions. Crucially, the absence of pre-stimulus effects supports the phasic hypothesis, suggesting metacontrol biases emerge dynamically rather than maintaining a stable tonic state. Correlational analyses showed task-specific and transient metacontrol biases. Persistence aligned with high-conflict demands in incongruent trials, while flexibility remained general across conditions. The lack of cross-task correlations between creativity and Flanker tasks further supports the phasic view, where biases adapt to immediate task requirements rather than representing stable traits. These findings align with reactive control theories, emphasizing transient, context-dependent neural adjustments. This study provides evidence for a phasic account of metacontrol, where cognitive control states dynamically shift to meet task-specific demands. This adaptive flexibility underscores the importance of real-time adjustments in human cognition.

**Supplementary Information:**

The online version contains supplementary material available at 10.1038/s41598-025-20479-8.

## Introduction

 A particularly important skill humans master is adapting to varying environmental and cognitive challenges. This ability is typically referred to as cognitive control. Traditional views of cognitive control emphasize persistence, which involves focusing on the task at hand, ignoring distractions, and maintaining concentration. Cognitive control is seen as crucial for tasks that require selectivity, such as when there is a lot of distracting information, cognitive control helps us stay focused on what’s relevant and ignore distractions. For instance, tasks that induce a high degree of response uncertainty by presenting distracting information, such as task-irrelevant flankers, are widely assumed to rely on and challenge cognitive control^1–8^. The idea is that cognitive control helps to increase the focus on task-relevant information and to gate out task-irrelevant information, allowing us to overcome distractions^9^. However, more recent theorizing has pointed out that this traditional understanding of cognitive control captures only one side of the control coin. In some tasks or environments, persistence is necessary (i.e., being highly focused, avoiding distractions, and putting in a lot of mental effort). For example, when solving a difficult problem or completing a task that demands precision, persistence in focus is crucial. In different situations, such as facing repeated failure or searching for creative solutions, require a difference approach. In these cases, maintaining rigid focus is counterproductive. Instead, shifting your focus, taking a step back, and thinking more broadly may be more helpful. Thus, being flexible – open to new ideas or different approaches – can help in overcoming challenges^10–12^. Therefore, cognitive control involves a dynamic balance between persistence (staying focused and selective) and flexibility (being open and adaptive), adapting to different situational demands.

### Metacontrol: balancing different processing modes

Authors have suggested that this balancing, which we refer to as metacontrol^11–15^, can functionally be described as regulating the degree to which the present action goal impacts ongoing decision-making and the degree to which alternative representations compete for selection. Simply speaking, metacontrol is about balancing how much you stick to your current goal (= persistence) versus how much you consider other options (= flexibility). Persistence is characterized by strong goal influence, meaning that the current goal dominates processing while alternative options are actively suppressed^12^. For instance, when solving a complex mathematical problem, a persistent strategy involves sticking with a particular method despite potential alternatives. Whereas the current goal can characterize flexibility has less influence, allowing alternative options to be considered with less competition^12^. Using the same mathematical problem example, if a method isn’t working, different approaches will be considered instead of insisting on the original one.

At the neural level, these two control modes are likely associated with distinct patterns of E/I (excitation/inhibition) balance. When adopting a persistency mode, the brain minimizes unnecessary activity and focuses only on what is essential. This means the E/I (excitation/inhibition) balance is shifted toward inhibition, reducing overall neural variability^16–20^. When adopting a flexibility mode, the brain engages in broader and more varied neural activity, exploring different options. This means the E/I balance is shifted toward excitation, increasing neural variability. Therefore, we argue that this neural regulation of cognitive control states suggests a potential marker of metacontrol biases, leading to the question of how we can investigate the neural activity of persistence vs. flexibility. One promising measure to capture this neural dynamic is the aperiodic exponent. Periodic neural activity is commonly assumed to subserve the signaling of information between brain modules, and different frequency bands are believed to be involved in different signaling loops^21,22^. Aperiodic activity^23^ refers to the neural activity that is not involved in frequency-based signaling and can thus be taken to be sensitive to the E/I ratio and reflect the degree to which the cognitive system is “neurally noisy”^16–20^. A higher aperiodic exponent indicates greater inhibition, leading to more stable and controlled neural activity, whereas a lower exponent suggests increased excitation and greater neural variability. Given this, the aperiodic exponent, which quantifies the aperiodic component of current neural activity^17,19,20,23^, might serve as an indicator of metacontrol biases. Indeed, there is increasing evidence found that the exponent increases (indicating less aperiodic activity and relative dominance of inhibitory over excitatory processes) under conditions that call for stronger metacontrol persistence but decreases under conditions that call for more metacontrol flexibility^16,18,24–26^. However, a critical question remains: does metacontrol operate as a stable trait (tonic) or a dynamic adjustment triggered by task demands (phasic)?

### Two possible scenarios for metacontrol

Accordingly, here, we were guided by two alternative scenarios: a “tonic view” and a “phasic view”. The key difference between the views is whether metacontrol biases are maintained as a stable neural state or if they emerge only when triggered by specific cognitive demands. In the tonic view, metacontrol biases are stable, long-lasting state that persist over time. Once a metacontrol state is activated, it remains relatively constant until a different one is required, such as flexibility. If the aperiodic exponent reflects metacontrol biases, we expect that specific conditions can induce a longer-lasting exponent change and stay on this level until another shift is needed. In contrast, the phasic view suggests that metacontrol is dynamic and stimulus-driven, adjusting based on immediate demands. Rather than maintaining an ongoing neural state of persistence or flexibility, metacontrol adjusts the way the incoming stimuli interact with cognitive processes in real time. This makes cognition more adaptive to changing situations. If so, the aperiodic exponent should only change after relevant stimuli appeared, rather than remaining continuously (i.e. not being adjusted depending what information has been encountered). The dual mechanisms of control theory^27^ distinguishes between proactive control, which involves sustained, goal-maintaining strategies, and reactive control, which is more flexible and stimulus-driven. Our study aims to test whether metacontrol operates as a tonic state (proactive control) or adapts dynamically to situational demands (reactive control). Recent findings support the phasic view, showing that the aperiodic exponent increases only after a relevant stimulus^20,28^. Thus, conditions that induce a metacontrol bias towards persistence or flexibility would not be invisible as a continuous shift in the aperiodic exponent but instead as a change in the phasic peak of the exponent occurring in response to task-relevant stimuli. To simplify, imagine listening to music where the volume only increases at key moments (phasic) rather than staying consistently loud or quiet (tonic). Similarly, the aperiodic exponent doesn’t stay shifted for long period, it changes in response to specific task-relevant stimuli.

### Strategy to examine the study’s questions

To investigate whether metacontrol biases are more consistent with the tonic or the phasic scenario, our study employed two manipulations aimed at inducing particular metacontrol biases: (1) A Flanker task with probability manipulations of congruent and incongruent trials: (2) Creativity tasks assessing divergent and convergent thinking to establish respective metacontrol states.

The *first manipulation* was established in a flanker task. In a flanker task, participants respond to a target symbol that appears in a particular location, which signals a left or right key-pressing response, while ignoring distracting flankers which can be congruent (same response as the target) or incongruent (opposite to the target response)^2,3^. Incongruent flankers are known to induce response conflict, a situation where the brain is momentarily unsure which response to make. To resolve this conflict and respond correctly to the target, you need to draw upon cognitive control processes^17,29–31^. For example, in the flanker task’s incongruent trials, if you’re supposed to press left for the target arrow but the surrounding flanker arrows pointing to the right, the brain then experiences conflict because the flankers push you toward an incorrect response. To get the correct answer, your cognitive control system must suppress the flankers’ influence and focus on the target. Incongruent trials are not only known to increase reaction time and error rates, but they also lead to an increase in the aperiodic exponent^17^. Interestingly, however, this increase does not seem to affect ongoing processes within the current trial but, rather, sets the stage for more effective processing in the next trial^17^. Hence, response conflict seems to trigger metacontrol adjustments, but these adjustments are apparently not targeted and not necessary for the solution of the present conflict. We manipulated the probability of congruent and incongruent trials to see if metacontrol biases remain stable (tonic) or only appear when needed (phasic). It is known that probability manipulations change the flanker effect and the way flankers are processed, in the sense that a high probability of congruent trials increases the effect size, whereas a low probability of congruent trials reduces it^4,32–34^. From a metacontrol perspective, this pattern can be characterized as indicating that a high probability of congruent trials biases metacontrol towards flexibility. If congruent trials are frequent, people get used to easy trials, adopting a flexibility bias, which shows fast responses but more interference when an incongruent trial appears. Whereas the high probability of incongruent trials biases metacontrol towards persistence. If incongruent trials are frequent, people prepare for more conflict, adopting a persistence bias, which shows slower responses but better resistance to interference. In the present experiment, we manipulated the probability of congruent/incongruent trials block-wise so that participants were encouraged to keep their metacontrol bias constant across the entire block.

According to the tonic scenario, the brain should establish and maintain a bias throughout the entire block, even before the stimuli appear. This would predict a higher aperiodic exponent in blocks with a high percentage of incongruent trials, and this effect should be visible both before and after stimulus presentation. According to the phasic scenario, the brain does not maintain a persistent state of control but instead reacts to each stimulus dynamically. This means that the aperiodic exponent would only increase after an incongruent stimulus appears, not before. There should be no pre-trial difference in the aperiodic exponent, meaning that participants do not prepare for incongruency in advance. This distinction is crucial because if the exponent increases only after the stimuli, it suggests metacontrol is phasic, whereas a pre-trial increase would suggest tonic. While the aperiodic exponent should be higher in incongruent than congruent trials, the interaction of this effect with the frequency manipulation is more difficult to predict. On the one hand, one might expect that preparation leads to a stronger bias towards persistence in incongruent trials (i.e., if people prepare for frequent incongruent trials, their persistence bias may become even stronger). This could lead to an even larger difference in the aperiodic exponent between congruent and incongruent trials in the block with more frequent incongruent trials. On the other hand, however, expectation might make frequent incongruent trials easier. If people expect that most trials will be incongruent, they may find them less demanding. This means that if these trials are frequent, the aperiodic exponent should be smaller for incongruent trials. This would mean that the aperiodic exponent wouldn’t increase as much for incongruent trials in the block with more frequent incongruent trials because participants are already prepared. In any case, however, the phasic scenario would predict an interaction between frequency and congruency, but this interaction should be restricted to the time after stimulus presentation.

The *second manipulation* we used to induce metacontrol biases was creativity tasks. Creativity falls into at least two different skills: divergent thinking, like in a brainstorming task, and convergent thinking, like when thinking “things through”^35^. Like other studies^36–41^, we used the Alternate Uses Task (AUT) to assess divergent thinking, and the Remote Associates Task (RAT) to assess convergent thinking. These two tasks can be assumed to imply different metacontrol biases; when solving AUT problems, the ability to switch between different ideas quickly is required, in this case promotes flexibility; when working on RAT quests, the ability to stay focused on solving a challenging problem is required, in this case promotes persistence. There is indeed converging evidence that they benefit in different if not opposite ways from conditions that are likely to induce flexibility and persistence biases^12^. In the present study, we wanted to test whether the individual bias obtained in the creativity tasks predicts the bias in the flanker task. A phasic scenario would not suggest such a relationship because the phasic, condition-specific changes in the aperiodic exponent should be much more task-specific. A tonic scenario, however, might suggest that people have particular styles or preferences with regard to their longer-term metacontrol states should stay consistent across tasks. If so, when people show a big difference in aperiodic exponents between the AUT and RAT (i.e., very flexible in AUT, very persistent in RAT) should also show a big difference in their metacontrol states when performing Flanker task blocks with different incongruency probabilities. This would indicate that they carry over their metacontrol biases across different tasks, supporting the tonic scenario. If the relationship between creativity tasks and the flanker task should be weak or nonsexist, this should support phasic view because metacontrol adjustment is momentary, stimulus-triggered and do not generalize across tasks.

## Results

### Behavior

The rated AUT scores exhibited good reliability (Intraclass correlation coefficient = 0.82). The descriptive statistics of all the indices are presented in Table 1.

**Table 1:** Descriptive statistics of behavioral data.

Indices	M	SD	Min	Max	Skew	Kurtosis
RAT	7.13	3.35	1.00	14.00	0.17	− 0.93
AUT	1.16	0.32	0.40	1.80	− 0.19	− 0.20
RT (FT_inc_ Congruent trials)	354.15	42.20	287.44	469.80	0.78	0.69
RT (FT_inc_ Incongruent trials)	376.61	46.37	299.38	506.75	0.94	0.54
RT (FT_con_ Congruent trials)	323.18	42.12	249.75	413.05	0.43	− 0.38
RT (FT_con_ Incongruent trials)	401.76	47.22	311.52	535.88	0.70	0.47
Error Rate (FT_inc_ Congruent trials)	0.06	0.05	0.00	0.19	1.20	1.30
Error Rate (FT_inc_ Incongruent trials)	0.08	0.06	0.01	0.29	1.11	1.70
Error Rate (FT_con_ Congruent trials)	0.03	0.02	0.00	0.10	1.18	1.31
Error Rate (FT_con_ Incongruent trials)	0.21	0.13	0.03	0.54	0.54	− 0.16

**Fig. 1:**
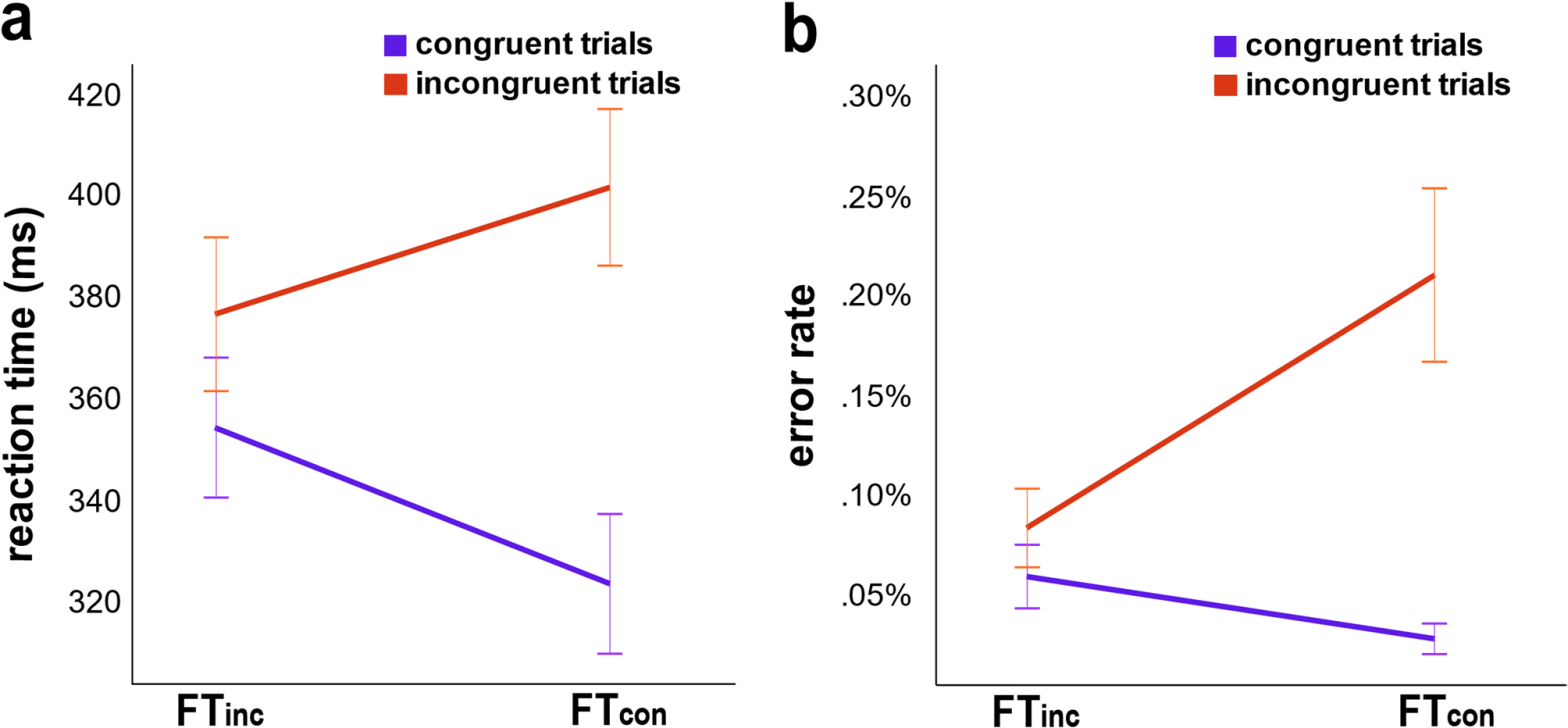
(a) Reaction time of FT_con_ and FT_inc_ conditions in congruent (purple line) and incongruent (orange line) trials. (b) Error rate of FT_con_ and FT_inc_ conditions in congruent and incongruent trials. Error bars represent standard deviations.

To examine the behavioral performance in the Flanker task, a 2 × 2 repeated-measures ANOVA of Congruency and Frequency (Fig. 1) was conducted. The main effect of Congruency was significant (F(1,37) = 217.67, *p* <.001, ηp2 = 0.86), with participants responding faster in congruent trials (339ms ± 7ms) than in incongruent trials (389ms ± 7ms), while the main effect of Frequency was not (F(1,37) = 1.44, *p* =.238, ηp2 = 0.04). Most importantly, the Congruency × Frequency interaction was significant (F(1,37) = 246.19, *p* <.001, ηp2 = 0.87), indicating that the congruency effect was substantially smaller in FTinc blocks (22.46ms ± 3.19ms) than in FTcon blocks (78.59ms ± 4.44ms, *p* <.001) as shown in the Fig. 1.

The same ANOVA on the error rates revealed significant main effects of Congruency (F(1,37) = 72.90, *p* <.001, ηp2 = 0.66), with higher error rates in incongruent trials (14.6% ± 1.5%) than in congruent trials (4.2% ± 0.5%), and of Frequency (F(1,37) = 41.14, *p* <.001, ηp2 = 0.53), with higher error rates in FTcon (11.8% ± 1.2%) than in FTinc blocks (7% ± 0.8%). Importantly, the significant Frequency × Congruency interaction (F(1,37) = 82.75, *p* <.001, ηp2 = 0.69), showed that the congruency effect was substantially smaller in FTinc blocks (0.02% ± 0.01%) than in FTcon blocks (0.18% ± 0.02%, *p* <.001) as shown in the Fig. 1.

### Aperiodic exponent

**Fig. 2:**
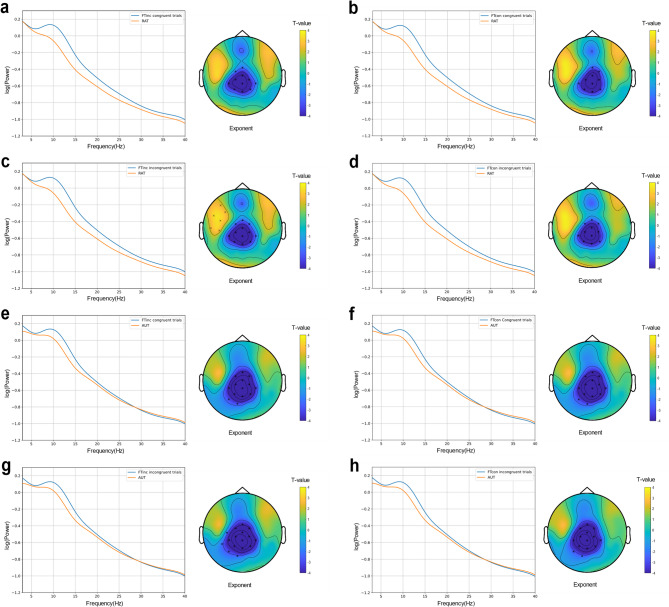
Scalp topography of t test between tasks and log–log transformed PSD plot after averaging all electrodes and participants in pre_stim intervals. (a) FT_inc_ congruent trials and RAT. (b) FT_con_ congruent trials and RAT. (c) FT_inc_ incongruent trials and RAT. (d) FT_con_ incongruent trials and RAT. (e) FT_inc_ congruent trials and AUT. (f) FT_con_ congruent trials and AUT. (g) FT_inc_ incongruent trials and AUT. (h) FT_con_ incongruent trials and AUT.

**Fig. 3:**
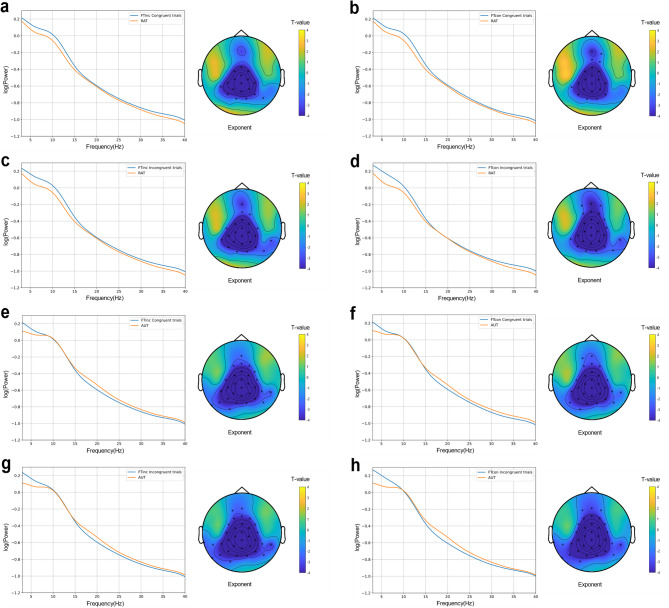
Scalp topography of t test between tasks and log–log transformed PSD plot after averaging all electrodes and participants in post_stim intervals. (a) FT_inc_ congruent trials and RAT. (b) FT_con_ congruent trials and RAT. (c) FT_inc_ incongruent trials and RAT. (d) FT_con_ incongruent trials and RAT. (e) FT_inc_ congruent trials and AUT. (f) FT_con_ congruent trials and AUT. (g) FT_inc_ incongruent trials and AUT. (h) FT_con_ incongruent trials and AUT.

**Fig. 4:**
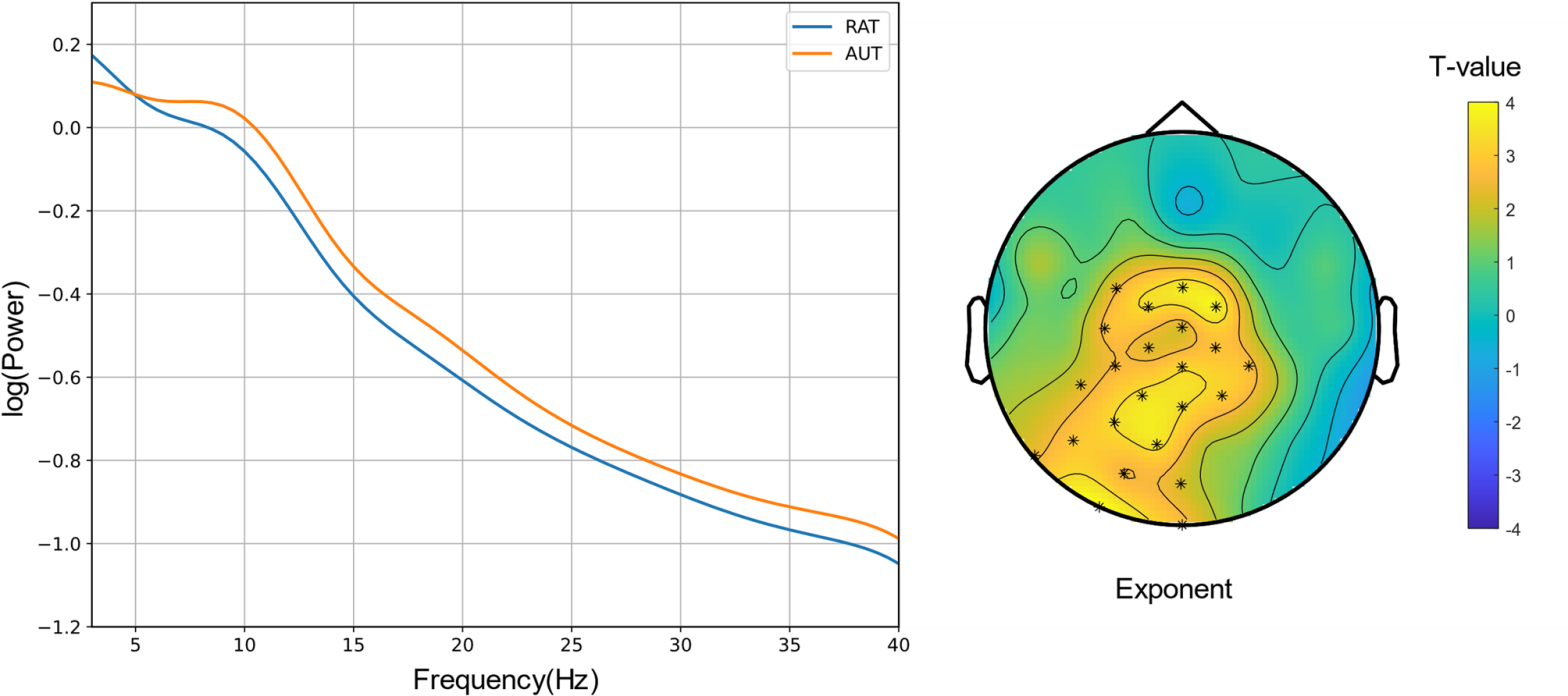
Scalp topography of t test between RAT and AUT and log–log transformed PSD plot after averaging all electrodes and participants in post_stim intervals.

**Fig. 5:**
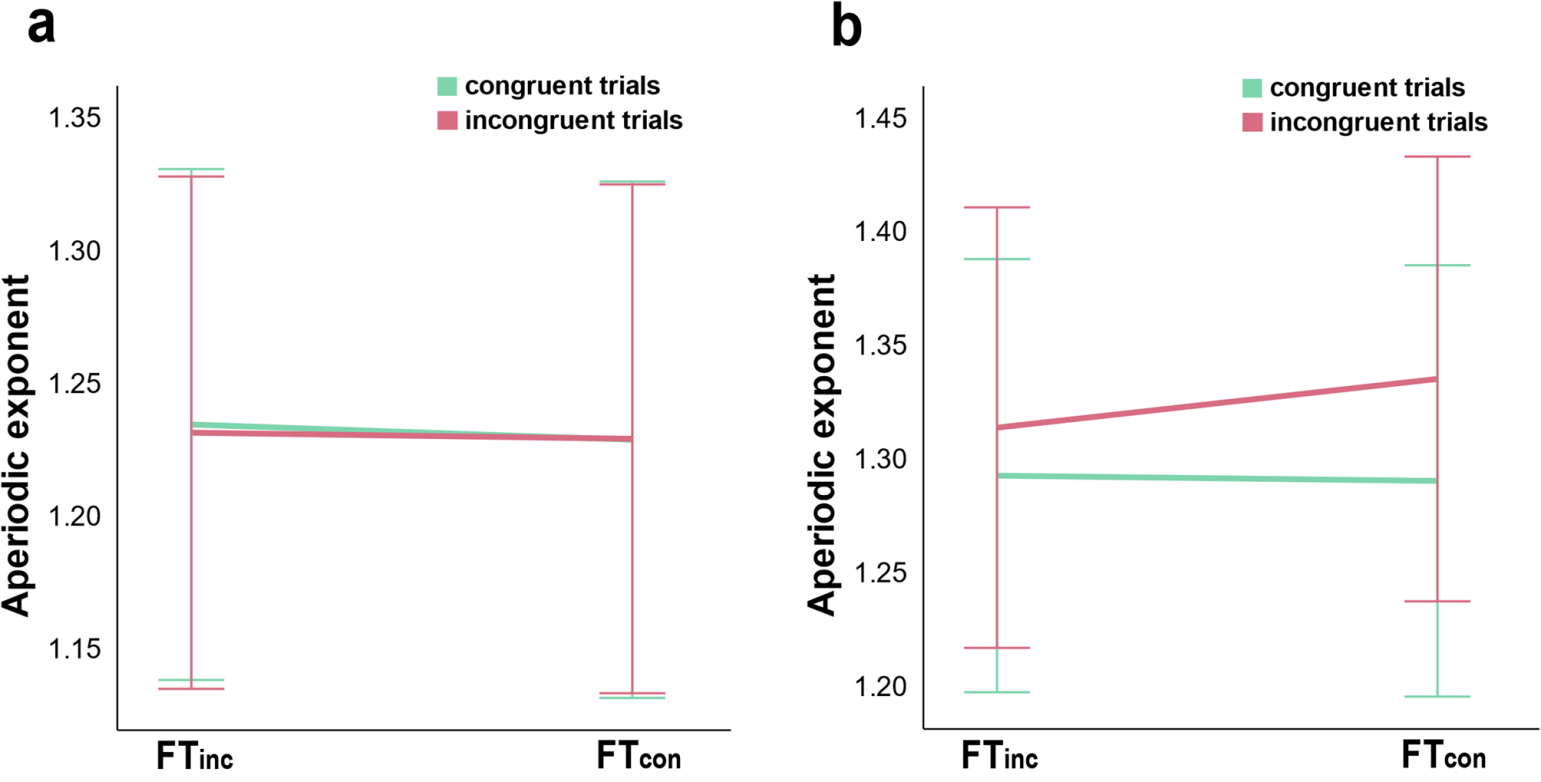
**(a)** Pre-stim exponent of FT_inc_ and FT_con_ conditions in congruent and incongruent trials. (b) Post-stim exponent of FT_inc_ and FT_con_ conditions in congruent and incongruent trials.

To explore the neural correlates of metacontrol, we analyzed the aperiodic exponent using a 2 × 2 × 2 repeated-measures ANOVA with Time (pre-stim, post-stim), Frequency (FTinc, FTcon), and Congruency (congruent trials, incongruent trials) as factors. Figure 2 illustrates the scalp topography and power spectral densities (PSD) for pre-stim intervals, while Fig. 3 presents post-stim data. Figure 4 compares RAT and AUT conditions, with Fig. 5 highlighting significant effects in the aperiodic exponent.

Significant main effects were found for Time (F (1,37) = 152.060, *p* <.001, ηp2 = 0.804), with a higher exponent after stimulus presentation (1.308 ± 0.047) than before (1.231 ± 0.047), and for Congruency (F (1,37) = 34.420, *p* <.001, ηp2 = 0.482), with higher exponents in incongruent trials (1.277 ± 0.047) than in congruent trials (1.262 ± 0.047), while the effect for Frequency was not significant (F (1,37) = 0.062, *p* =.804, ηp2 = 0.002).

All three two-way interactions were significant: Time × Frequency (F (1,37) = 15.202, *p* <.001, ηp2 = 0.291), Time × Congruency (F (1,37) = 39.736, *p* <.001, ηp2 = 0.518), and Frequency × Congruency (F (1,37) = 4.954, *p* =.032, ηp2 = 0.118), as was the three-way interaction of Time × Frequency × Congruency (F (1,37) = 7.666, *p* =.009, ηp2 = 0.172. To disentangle the latter, two 2 × 2 repeated-measures ANOVA of Frequency and Congruency were conducted separately for the pre-stim and the post-stim intervals. In the analysis of the pre-stim data, no effect was significant (Frequency: F (1,37) = 0.131, *p* <.719, ηp2 = 0.004; Congruency: F (1,37) = 0.193, *p* <.663, ηp2 = 0.005; Congruency × Frequency: F (1,37) = 0.228, *p* <.636, ηp2 = 0.006). The analysis of the post-stim data showed a significant main effect of Congruency was significant (F (1,37) = 55.081, *p* <.001, ηp2 = 0.598), with lower exponents in congruent trials (1.292 ± 0.047) than in incongruent trials (1.325 ± 0.048), and a significant Congruency × Frequency interaction (F (1,37) = 11.379, *p* =.002, ηp2 = 0.235)—due to a larger congruency effect in the blocks with frequent congruent trials.

A paired sample T test contrasting the FOOOF exponents in RAT and AUT confirmed that the exponent was significantly higher in RAT (M = 1.25, SD = 0.28) than in AUT (M = 1.19, SD = 0.27); *t* (37) = 2.05, *p* =.048, Cohen’s *d* = 0.33.

### Correlations between tasks

We conducted non-parametric tests to examine the relationships between RAT/AUT and Flanker conditions for pre-stim (Table 2) and post-stim (Table 3) intervals. For the pre-stim intervals, the comparison between the correlations of RAT and FTinc and the correlations of RAT and FTcon did not show significant differences for congruent trials. However, for the incongruent trials, the correlations of RAT and FTinc were higher than the correlations of FTcon. The comparison between the correlations of AUT and FTinc and the correlations of AUT and FTcon showed significant differences for congruent trials as well as for incongruent trials. For the post-stim intervals, the results displayed the same pattern as the pre-stim intervals.

**Table 2:** Comparisons between correlation pairs of RAT/AUT with flanker task conditions for pre-stim intervals.

Correlational Pairs Comparison		SD	Median	Z	*p*
1	RAT and FTinc Congruent trials vs. RAT and FTcon Congruent trials	0.173 0.209	0.945 0.888	−1.318	0.188
2	RAT and FTinc Incongruent trials vs. RAT and FTcon Incongruent trials	0.188 0.213	0.930 0.893	−3.821	< 0.001
3	AUT and FTinc Congruent trials vs. AUT and FTcon Congruent trials	0.183 0.214	0.865 0.764	−4.608	< 0.001
4	AUT and FTinc Incongruent trials vs. AUT and FTcon Incongruent trials	0.181 0.211	0.840 0.756	−4.719	< 0.001

**Table 3:** Comparisons between correlation pairs of RAT/AUT with flanker task conditions for post-stim intervals.

Correlational Pairs Comparison		SD	Median	Z	*p*
1	RAT and FTinc Congruent trials vs. RAT and FTcon Congruent trials	0.167 0.206	0.901 0.876	−1.531	0.126
2	RAT and FTinc Incongruent trials vs. RAT and FTcon Incongruent trials	0.174 0.184	0.928 0.866	−4.557	< 0.001
3	AUT and FTinc Congruent trials vs. AUT and FTcon Congruent trials	0.179 0.202	0.814 0.730	−5.021	< 0.001
4	AUT and FTinc Incongruent trials vs. AUT and FTcon Incongruent trials	0.165 0.189	0.797 0.738	−4.873	< 0.001

### Correlations between effect sizes

To examine associations between the effect sizes derived from the RAT and AUT and the effect sizes from the FTinc and FTcon conditions for both congruent and incongruent trials using Spearman’s correlation analysis. Separate analyses were performed for pre-stim and post-stim intervals. For the pre-stim intervals, RAT-AUT did not correlate significantly with the FTinc – FTcon for either congruent trials (*rho* = − 0.300, *p* =.068) or incongruent trials (*rho* = − 0.057, *p* =.735). Similarly, in the post-stim intervals, no significant correlations were found between the RAT – AUT and FTinc – FTcon for congruent trials (*rho* = − 0.197, *p* =.236) or incongruent trials (*rho* = − 0.028, *p* =.866). However, significant positive correlations were observed between the FTinc – FTcon congruent trials and FTinc – FTcon incongruent trials. This was observed in both the pre-stim intervals (*rho* = 0.618, *p* <.001) and the post-stim intervals (*rho* = 0.724, *p* <.001), indicating a consistent relationship between congruent and incongruent trials within different time window.

## Discussion

The aim of this study was to see whether metacontrol is more likely to operate according to a tonic or a phasic scenario. The tonic scenario suggests that a particular metacontrol bias is established by creating a particular neural state that governs the information processing style as long as this state exists. The phasic scenario, in turn, suggests that metacontrol biases are mainly represented by parameterizing the operations that are triggered by a particular stimulus. Accordingly, metacontrol biases would not necessarily be accompanied by particular neural states, but they would rather determine the characteristics of states that are involved in processing a particular stimulus and/or task. The aperiodic exponent was used as a neural indicator of metacontrol shifts as done in previous studies^20,28^. We assumed that metacontrol biases would come with changes in the aperiodic exponent so that this exponent and changes therein would serve as indicators of the dynamics of metacontrol-related states.

Our findings show that our versions of the three tasks we used successfully replicated previous observations^41–43^, confirming that participants adjusted their cognitive control strategies based on task demands. The behavioral findings produced the well-known flanker effect, with better performance in congruent than in incongruent trials, and the previously observed reduction of the congruency effect with a high frequency of incongruent trials^2,4,44–48^, possibly due to a persistence bias that helps resisting distractions. Specifically, a high frequency of incongruent trials (FT_inc_ blocks) encouraged a persistence bias. In this case incongruent trials were frequent, participants adapted by slowing down but reducing interference from conflict. In contrast, a high frequency of congruent trials (FT_con_ blocks) encouraged a flexibility bias. Because congruent trials were frequent, participants adapted by responding faster, but this made them more vulnerable to conflict when the less frequent incongruent trials appeared. This pattern implies that participants modulate their cognitive strategies according to task demands, favoring persistence in difficult contexts and flexibility when task conditions allow^33,49^.

Along the same lines, analyses of the aperiodic exponent replicated the previous finding that the more persistence-demanding incongruent condition comes with a higher exponent than the congruent condition^11,17,20,24,25,31^. Variation in the magnitude of the aperiodic exponent is thought to reflect variations in neural inhibition, which helps maintain cognitive focus^23^. During high-conflict conditions (incongruent trials), the brain increases inhibition to suppress distractions, leading to a higher aperiodic exponent and a more controlled and stable state^9^. Our ANOVA tests of the FOOOF exponent yielded a significant interaction of Congruency × Time × Frequency, where incongruent trials showed higher exponents after stimulus onset than congruent trials. This means that neural inhibition increased in response to incongruency, helping to enhance persistence. Notably, the lack of significant effects in pre-stimulus data (i.e. before a stimulus was presented that is of behavioral relevance) suggests that metacontrol adjustments do not establish a lasting, tonic state but rather emerge in response to task stimuli, supporting the phasic hypothesis. This aligns with previous research demonstrating that cognitive control is not a static trait but rather adapts to situational requirements^11,20,27^. For instance, according to the proactive and reactive control theory^27^, proactive control is described as sustained, goal-maintaining cognitive control that remains active over time, which aligns with the tonic view. Reactive control is more flexible and stimulus-driven, triggered by specific stimuli as needed, which is in line with the phasic view. The observed post-stimulus aperiodic exponent increase during incongruent trials is consistent with increased persistence demands. Since metacontrol adjustments were only observed post-stimuli, this fits the definition of reactive control, meaning that participants adapted their cognitive state only when necessary rather than maintaining a stable mode. Moreover, Zhang et al.^20^ found that the aperiodic exponent increased in response to cognitive conflict, similar to our results in incongruent trials of the Flanker task. These convergent findings reinforce the idea that metacontrol biases emerge as transient, context-dependent states, extending the dual mechanisms of control framework^27^ into the domain of neural dynamics.

The significant Congruency × Frequency interaction observed in post-stimulus intervals provides further evidence that participants adapt their metacontrol bias in response to specific trial types, dynamically adjusting between persistence and flexibility depending on the immediate demands^50^. Specifically, incongruent trials elicited higher aperiodic exponents in FTinc blocks, where persistence is advantageous, indicating enhanced cognitive control under conflict. This pattern was not evident before the stimulus presentation, underscoring the reactive nature of these neural adjustments rather than being a pre-set, stable state. Our findings consistent with Zhang^20^, who reported changes in EEG activity during high-conflict cognitive tasks. Pi^28^ linked aperiodic activity primarily to resting-state cognitive control styles, highlighting the potential for dynamic, task-driven modulation of neural states. These results contribute to the growing body of literature suggesting that metacontrol is not a stable trait but rather a transient, adaptive response to situational demands. By demonstrating the phasic nature of aperiodic exponent changes, our study advances the understanding of how neural inhibition and excitation balance dynamically support cognitive flexibility and persistence. The non-parametric tests were conducted to examine the differences in the correlations between RAT/AUT and FTinc/FTcon for pre-stim and post-stim intervals, respectively. The results indicate a consistent pattern across both pre-stim and post-stim, with significant differences in correlations between RAT/AUT and FTinc/FTcon, which further support the phasic view. Specifically, only in incongruent trials the correlation between RAT (persistence) and FTinc was higher than between RAT and FTcon. This suggests that persistence is particularly relevant in high-conflict conditions (FTinc incongruent trials), where strong cognitive control is needed to resolve response conflict^4,9^. For AUT, the correlation with FTinc was consistently higher than with FTcon across both congruent and incongruent trials, indicating that flexibility is broadly relevant in FTinc blocks, regardless of trial type. Overall, these findings demonstrate that persistence bias is selectively associated with the high-conflict context, whereas flexibility supports adaptation in FTinc more generally, rather than being tied to specific trial type. These dynamic, phasic adjustments may allow for more efficient adaptation to task-specific requirements, balancing persistence and flexibility in need^51,52^.

Spearman’s correlation analysis revealed that the metacontrol biases derived from the creativity tasks did not correlate significantly with the biases from the Flanker task in either congruent or incongruent trials across both pre-stim and post-stim intervals. This finding contradicts the tonic view, which predicts that metacontrol biases should be stable across different tasks. However, this pattern supports more of the phasic view, in which the metacontrol biases are not expected to manifest like a stable, trait-like state across different tasks but like a transient, task-specific state adjusted to specific task demands^11,27,53^. Moreover, a significant correlation was observed between FTinc – FTcon congruent and incongruent trials across both pre-stim and post-stim intervals. This indicates that metacontrol biases were consistent within the flanker task but did not generalize across functions, reinforcing the phasic view. These results have broader implications for understanding how metacontrol might operate in real-world settings where cognitive demands fluctuate. The phasic model of metacontrol could help explain how individuals adapt their focus and cognitive strategies in dynamic environments, such as during complex problem-solving or multitasking scenarios. Overall, these findings imply that metacontrol is not a stable cognitive trait but dynamically adjust to the immediate context of a specific task, and its context is dependent instead of maintained across different tasks^9,10,51^. Additionally, we conducted Pearson correlation analyses between the exponent and behavioral performances for the pre-stimulus data and for the post-stimulus data. Results showed that no significant correlations were found. The absence of significant correlations between the aperiodic exponent and behavioral performance measures further supports this phasic account, suggesting that the exponent reflect momentary adjustments rather than consistent, stable cognitive traits.

In summary, our findings support a phasic perspective of metacontrol, emphasizing that cognitive control states are dynamic and transient to meet situational demands rather than being sustained as stable, long-term states. The phasic view better accounts for naturalistic human cognition. In real life, cognitive resources like attention and control are allocated and adjusted in real time based on the current goal instead of maintaining the same state. While our study provided insights into the nature of metacontrol biases, some limitations remain. First, our study focused on the general aperiodic exponent which may have obscured local neural effects. Future study could employ more localized analyses to explore specific brain regions involved in metacontrol processes. Additionally, while our study focused on cognitive control and creativity tasks, extending this research to other cognitive domains and diverse populations could enhance the generalizability of our findings. Finally, exploring metacontrol dynamics in clinical populations could provide valuable insights into how adaptive cognitive control mechanisms might be leveraged in therapeutic contexts. Future research should also investigate the dynamics further using a paradigm tailored to this goal. It is also worth noting that recent work has highlighted aperiodic broadband power, which reflects both the slope and intercept, as another potential marker of cognitive demand^54,55^. Although our analyses focused on the exponent, future studies could take aperiodic broadband power into consideration to provide a more comprehensive picture of metacontrol dynamics.

## Methods

### Participants

The sample in this study was also used in a previous study^34^, which examined the neurophysiological dynamics of metacontrol states. We initially recruited 43 native German-speaking participants via advertisement. Out of these, five participants were excluded due to poor behavioral performance (*N* = 3), EEG data quality (*N* = 1), and withdrawal from the study (*N* = 1). The final sample comprised 38 participants (24 females; Mean age = 29.82, SD = 10.90). Prior to participation, all participants were screened, and no psychiatric or neurological disorders were reported. All participants provided a written informed consent form before the study procedure and were remunerated either through course credit or financial compensation after completion. This study received approval from the ethics committee of the Faculty of Medicine of TU Dresden, and all relevant ethical regulations for human research were followed.

### Task procedures

To investigate our study question, we used the Remote Associates Test (RAT) to induce a metacontrol bias toward persistence, and the Alternative Uses Task (AUT) to induce a metacontrol bias toward flexibility. To do the same in the Flanker task, we manipulated the frequency of congruent and incongruent trials. A higher frequency of incongruent trials was used to induce a persistence bias, and a higher frequency of congruent trials to induce a flexibility bias. Participants performed all the tasks (supplementary Fig. 1) in two sessions. In one session, RAT and AUT were presented, and in another session, the Flanker Task was presented. The order of the two sessions and the tasks within a session were randomized. All tasks were programmed and presented using Presentation software (https://www.neurobs.com/).

### RAT

In the RAT, participants were presented with three stimulus words, for instance, “Geheimnis-Note-Konto” (e.g., the German words corresponding to “secret” – “note” – “account”; refer to Supplementary Table S1 for the complete list). The task required participants to generate a fourth word (e.g., “Bank”) that, when combined with each of the three stimulus words, formed a common compound word (e.g., Bankgeheimnis-Banknote-Bankkonto). The 20 triads used in the experiment were selected from a German version of the RAT^56^. Stimuli were displayed centrally on the screen, with each trial beginning with the appearance of a fixation cross for two seconds. Participants were given a maximum of one minute to generate a solution. Upon identifying a solution, participants indicated their response via a keypad, after which they entered their answer. If participants could not produce an answer within the time limit, no input was required.

### AUT

In the AUT, participants were presented with words denoting common objects, such as “leere Konservendose” (e.g., the German word for “empty can”; see Supplementary Table S2 for the full list of objects). Of the 20 objects used, 4 were taken from the German version of AUT^57^, while the remaining 16 were derived from the Chinese version of AUT^40,58^ and subsequently translated into German by a native speaker. Participants were instructed to generate creative uses for each object. Following the idea generation phase, they were required to submit their most creative response by typing it on a keyboard. The stimuli were presented centrally on the screen. Each trial began with the display of a fixation cross for two seconds, followed by the object name, which remained on the screen for one minute. Once the one-minute presentation period had elapsed and the participant’s response was recorded, the next trial began.

### Flanker task

We adapted the Flanker task, a paradigm previously used by our research group^59–61^, to induce a metacontrol bias towards persistence or flexibility. We manipulated these biases by varying the frequency of congruent and incongruent trials. Specifically, a 70:30 ratio of incongruent trials vs. congruent trials (FT_inc_) was used to induce a persistence bias, while a 30:70 ratio of incongruent trials vs. congruent trials (FT_con_) was used to induce flexibility. In FT_inc_ blocks, incongruent trials comprised 70% of the total, and congruent trials 30%. Alternatively, in FT_con_ blocks, congruent trials made up 70% of the total, and incongruent trials 30%. The task was shown on a 20-inch TFT screen, and the participants sat at a distance of approximately 50 cm from the screen. In each trial of the task, a jittered fixation cross was presented first, with a duration of 700-1100ms. Subsequently, the presentation of the two Flanker stimuli (200ms) was followed by the Target stimulus for 300ms (stimulus onset asynchrony = 200ms). The Flanker and Target stimuli were displayed vertically in the middle of the screen. After the onset of the Target stimulus, participants were asked to indicate the direction of the Target pointing within 650ms by pressing the corresponding left or right control key. After the response, the Flanker and Target stimuli disappeared simultaneously. In the congruent trials, both Flanker and Target arrows were aligned, pointing in the same direction. This alignment would facilitate a clear response. Conversely, in the incongruent trials, the Target arrow is oriented in an opposing direction to the Flanker arrows, thereby creating a response conflict. This conflict environment was intended to assess participants’ ability to disregard the interfering Flanker stimuli and focus exclusively on the direction of the Target stimulus. This design enables us to investigate participants’ capability of resolving cognitive conflicts and adapt their responses in the presence of conflicting information.

### EEG data acquisition and pre-processing

While participants performed the task, EEG signals were recorded with 60 equidistant Ag/AgCl electrodes embedded in an elastic cap (EasyCap Inc.). For the recording, the software BrainVision Recorder 2.1 and BrainAmp amplifiers (Brain Products GmbH, Gilching, Germany) were used. The reference electrode was placed at the coordinates of theta = 90, phi = 90, and the ground electrode at theta = 58, phi = 78. The sampling rate was set to 500 Hz, and electrode impedances were kept below 10 kΩ. The recorded data were pre-processed offline using the Automagic toolbox^62^ and EEGLAB toolbox^63^ on MATLAB R2022a (The MathWorks Corp.). The sampling rate of raw EEG signals was reduced to 256 Hz, and flat channels were removed before re-referencing the data to an average reference. We then applied the PREP preprocessing pipeline^64^ and the EEGLAB ‘clean_rawdata()’ pipeline, which removed 50 Hz line noise and excluded contamination by bad channels on the average reference. A finite impulse response high-pass filter (0.5 Hz, order 1286, stop-band attenuation − 80 dB, transition band 0.25–0.75 Hz) was employed to identify and remove channels of flat-line, noisy, and outlier. To remove electromyographic artifacts, a 40 Hz (sinc finite impulse response filter; order: 86) low-pass filter was applied^65^. Electro-oculographic artifacts were removed using subtraction^66^. Subsequently, Independent Component Analysis was performed to detect and exclude muscle, cardiac, and remaining ocular artifacts using the Multiple Artifact Rejection Algorithm^67,68^. For RAT and AUT, the entire problem-solving or ideation phase was considered to reflect a general cognitive state (convergent or divergent thinking, respectively). Accordingly, continuous EEG signals of RAT/AUT tasks were segmented into consecutive 1000ms epochs to capture ongoing neural activity across the full trial for power spectral density (PSD) calculation. For the Flanker task specifically, the continuous EEG signals were segmented into pre- and post-stim epochs, respectively. Pre-stim data was segmented as 1000ms before the Flanker distractors (e.g., where the upper and lower triangles are present). Post-stim data was segmented as 1000ms after the Flanker distractors.

### Parameterization of the spectral data

The Power Spectral Density (PSD) was calculated for each participant and electrode using Welch’s method, as implemented in MATLAB with a 0.25-second Hamming window and 50% overlap. Pre-stim PSD estimates were derived for the time interval from 1000 milliseconds before the presentation of the Flanker stimulus to 0. Post-stim PSD estimates were derived for the time interval from 0 to 1000 milliseconds following the presentation of the Flanker stimulus. The FOOOF (Fitting Oscillations and One Over *f*) Python toolbox (version 1.0.0; https://fooof-tools.github.io/fooof/) was utilized to decompose the power spectra into periodic and aperiodic components^17,20^. This method effectively extracts periodic and aperiodic components within the power spectra^23^. For the present study, the aperiodic exponent was extracted from frequencies ranging between 3 and 40 Hz, consistent with methodologies employed in previous research^17^. The FOOOF analysis was configured with the following parameters: aperiodic mode = “fixed”, peak width limits = [1, 8], the maximum number of peaks = 8, minimum peak height = 0.05, default settings otherwise. The power spectra fitting procedure was applied to each electrode, participant, task condition, and time period. The average R² value for the spectral fits across all participants was 0.978.

## Statistical analysis

### Behavior

In the present study, responses to RAT were evaluated according to the standards by Landmann^56^. Specifically, correct responses were awarded a score of 1, while incorrect responses were assigned a score of 0. For the AUT, two native German-speaking judges (mean age = 33.5 years, 1 female) were recruited to assess the creativity of participants’ responses. Creativity was rated on a scale ranging from 0 (“not creative at all”) to 3 (“very creative”). The intra-class correlation coefficient (ICC) was calculated to assess the reliability of the judges’ ratings. The final creativity score for each response was determined by averaging the ratings provided by the two judges. The behavioral data for the Flanker task were analyzed using SPSS Version 29.0. The 2 × 2 repeated-measures ANOVA was performed on the error rates (ER) and reaction time (RT). The within-subject factors included Frequency (FTinc vs. FTcon) and Congruency (congruent trials vs. incongruent trials).

### Aperiodic exponent

The exponents of the aperiodic signal were derived for each participant and EEG channel using the FOOOF (Fitting Oscillations & One Over F) method. This resulted in a 60 × 38 matrix (channels × participants). First, we conducted analysis at the ‘global’ level. Before statistical analysis, data from all scalp electrodes were averaged for each participant to create a ‘global’ exponent, representing a more general aperiodic measure across the scalp. This approach was adopted since no priori assumptions about how the aperiodic signal varies across the scalp^17,20,69,70^. A 2 × 2 × 2 repeated-measures ANOVA of Time (pre-stim, post-stim), Frequency (FTinc, FTcon), and Congruency (congruent trials, incongruent trials) on the ‘global’ exponent was performed. We also conducted two 2 × 2 repeated-measures ANOVA of Frequency (FTinc, FTcon) and Congruency (congruent trials, incongruent trials) for pre-stim and post-stim, respectively.

### Correlations between tasks

In addition to the similarities between functionally equivalent control states, we also examined the differences between the correlations of RAT/AUT and different Flanker conditions. Specifically, Pearson correlations were computed at the channel level between the RAT exponents and all Flanker task conditions’ exponents and between the AUT exponents and all Flanker task conditions’ exponents. The resulting correlation coefficients were then Fisher Z-transformed to normalize their distribution. Finally, the Z-transformed coefficients were compared using the non-parametric Wilcoxon Signed-Rank Test to assess differences between the correlation pairs.

### Correlations between effect sizes

We first computed the individual effect sizes for each task to examine whether the manipulation in the creativity tasks is associated with the corresponding manipulation in the Flanker task. Specifically, we calculated the difference in aperiodic exponents between the RAT and AUT tasks to represent each participant’s metacontrol bias in the creativity tasks. Similarly, we computed the difference in aperiodic exponents between FTinc and FTcon conditions in the Flanker task, separately for congruent and incongruent trials, to assess metacontrol biases under different response conflicts. These effect sizes were computed for both the pre-stim and post-stim intervals. We then used Spearman’s correlation to assess associations between the effect sizes obtained from RAT – AUT and those from FTinc – FTcon conditions for congruent trials and incongruent trials.

## Supplementary Information

Below is the link to the electronic supplementary material.

Supplementary Material 1

## Data Availability

Data supporting the article can be found in OSF [https://osf.io/ta42r]. Further material can be obtained from the corresponding author upon reasonable request.
