# Supplementary material for: Metacontrol is reflected in phasic but not tonic cognitive control dynamics
Source: Sci Rep. 2025 Sep 24;15:32699. doi: 10.1038/s41598-025-20479-8 (PMC12460678; doi:10.1038/s41598-025-20479-8)
Supplement: Supplementary file 1 — Supplementary Material 1 [file 41598_2025_20479_MOESM1_ESM.docx]

**Supplementary Information**

***Supplementary Table 1*** *Remote Associates Task (RAT)*

RAT items were taken from a German version of RAT ^1^. The original version from Landmann includes 130 items in total. Based on the correct solution rate in 60s time interval, we took 20 items in the middle range (e.g., item from 37-56). And another 3 items as practice trials’ use. The order of all the items was randomized in the experiment. Table S1 shows the items in our study.

|  | Cues | Solution |
| --- | --- | --- |
| Practice 1 | Geheimnis – Note – Konto | Bank |
| Practice 2 | Pelz – Tasche – Schutz | Mantel |
| Practice 3 | Elch – Aids – Pilot | Test |
| Task 1 | Gewitter – Duft – Staub | Wolke |
| Task 2 | Heft – Titel – Internet | Seite |
| Task 3 | Joker – Kabel – Nummer | Telefon |
| Task 4 | Falte – Brett – Kleid | Bügel |
| Task 5 | Stirn – Achsel – Mund | Höhle |
| Task 6 | Deutung – Note – Tänzer | Traum |
| Task 7 | Dorf – Feld – Gipfel | Berg |
| Task 8 | Streit – Bruch – Versprechen | Ehe |
| Task 9 | Geist – Fahrt – Segel | Schiff |
| Task 10 | Wurm – Regal – Sendung | Buch |
| Task 11 | Anzug – Kapsel – Welt | Raum |
| Task 12 | Spiegel – Qualität – Röhre | Bild |
| Task 13 | Nase – Luft – Zange | Loch |
| Task 14 | Zeit – Meile – Bruch | Loch |
| Task 15 | Rose – Schere – Möbel | Garten |
| Task 16 | Wein – Körper – Tür | Glas |
| Task 17 | Gasse – Sitz – Müll | Sack |
| Task 18 | Ei – Müsli – Obst | Schale |
| Task 19 | Hütte – Futter – Steuer | Hund |
| Task 20 | Brille – Ochse – Jagd | Horn |

***Supplementary Table 2*** *Alternate Uses Task*

20 cue objects were used in our study. In which 4 were taken from the German version of AUT ^2^, 16 were taken from the Chinese version of AUT ^3,4^ and translated into German by native German speaker. The Table S2 shows the objects in our study. The first four are from Schoppe’s and rest are from Ding’s. The order of all the objects was randomized in the experiment.

|  | Cues |
| --- | --- |
| Object 1 | leere Konservendose |
| Object 2 | einfache Schnur |
| Object 3 | ein Ziegelstein |
| Object 4 | Schere |
| Object 5 | Zahnstocher |
| Object 6 | Teller |
| Object 7 | Kerze |
| Object 8 | Löffel |
| Object 9 | Kleber |
| Object 10 | Weinflasche |
| Object 11 | Haargummi |
| Object 12 | Tasse |
| Object 13 | Münze |
| Object 14 | Schachtel |
| Object 15 | Eiswürfel |
| Object 16 | Bleistift |
| Object 17 | Kleiderbügel |
| Object 18 | Ruder |
| Object 19 | Socke |
| Object 20 | Eierschale |

*
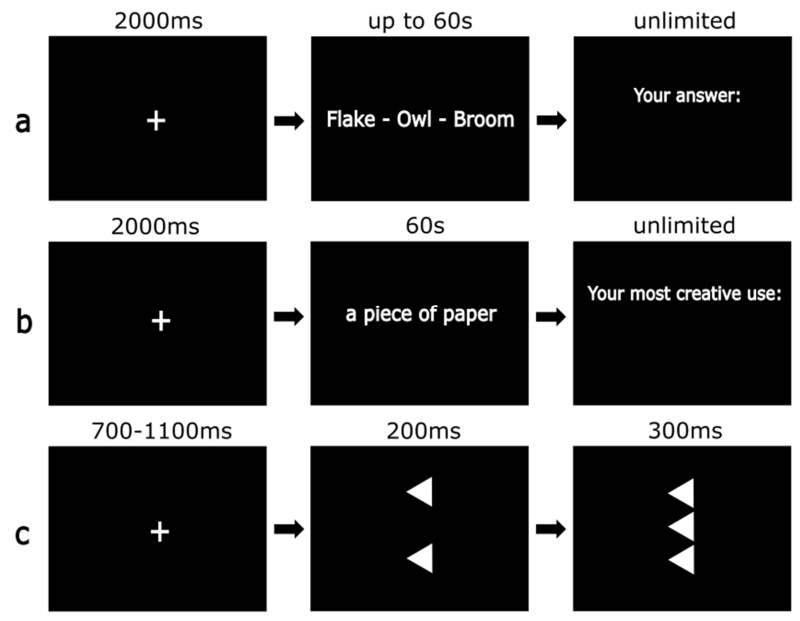
*

***Supplementary Figure 1*** *Depiction of the different tasks. a single trial procedure of the RAT task is shown. b single trial procedure the AUT task is shown. c single congruent trial procedure of the Flanker task is shown. For details on the tasks please refer to the text. Note that all the stimuli were presented in German during experimenting.*

**Supplementary References**

1. Landmann, N. *et al.* Entwicklung von 130 deutschsprachigen Compound Remote Associate (CRA)-Worträtseln zur Untersuchung kreativer Prozesse im deutschen Sprachraum. *Psychol. Rundsch.* **65**, 200–211 (2014).

2. Schoppe, K.-J. *Verbaler Kreativitäts-Test (V-K-T): Ein Verfahren Zur Erfassung Verbal-Produktiver Kreativitätsmerkmale : Handanweisung*. (Göttingen: Hogrefe, 1975).

3. Ding, K. *et al.* Recognizing ideas generated in a creative thinking task: Effect of the subjective novelty. *Curr. Psychol.* **42**, 529–541 (2023).

4. Wang, X. *et al.* The contribution of divergent and convergent thinking to visual creativity. *Think. Ski. Creat.* **49**, 101372 (2023).
